# Glycosylation of canine tetherin is essential for its antiviral activity against H3N2 canine influenza virus

**DOI:** 10.3389/fvets.2025.1641963

**Published:** 2025-07-23

**Authors:** Liang Xu, Yuwen Hou, Shizhe Liu, Yixin Dai, Jiajun Ou, Shaotang Ye, Zhen Wang, Gang Lu, Shoujun Li

**Affiliations:** 1 College of Veterinary Medicine, South China Agricultural University, Guangzhou, China; 2 Guangdong Technological Engineering Research Center for Pet, Guangzhou, China

**Keywords:** tetherin, N-linked glycosylation, mutation, canine influenza virus (CIV), antiviral activity

## Abstract

Tetherin is an interferon-induced-expressing transmembrane protein that utilizes a unique topology to restrict the release of enveloped viruses from the surface of the cell membrane. N-linked glycosylation plays an important role in protein post-translational modifications. To investigate the role of glycosylation in the antiviral activity of canine tetherin, its potential glycosylation sites were predicted and mutated, and the effects of glycosylation site mutations or treatment with a glycosylation inhibitor on the ability of canine tetherin to restrict H3N2 canine influenza virus (CIV) replication were examined. Mutations in the glycosylation sites of canine tetherin (N72A, N99A, and N72,99A) lead to changes in its intracellular distribution and weakened or even lost antiviral activity against H3N2 CIV. Similarly, the subcellular localization of tetherin after tunicamycin treatment was altered, and its antiviral activity was weakened. Colocalization analysis revealed that the colocalization of canine tetherin and H3N2 CIV protein was weakened under the condition of impaired glycosylation. These results indicate that canine tetherin maintains its localization in the cell membrane through glycosylation and exerts its antiviral activity. This study provides new insights into the antiviral mechanisms of host restriction factors and offers a theoretical basis for developing small-molecule anti-influenza strategies targeting glycosylation modifications.

## 1 Introduction

Tetherin is an innate immune factor which the antiviral activity was discovered in its ability to restrict the release of Vpu-deficient type I human immunodeficiency virus from the cell membrane surface (1, 2). Subsequent studies demonstrated that tetherin has the ability to restrict the release of a wide range of enveloped viruses (3). Previous studies have shown that the human tetherin CC domain contains two conserved asparagine (Asn) residue sites (N65 and N92) (4). Asn-linked glycosylation is a highly regulated post-translational modification that is important for the structure and function of eukaryotic proteins (5). N-linked glycosylation occurs primarily in the endoplasmic reticulum and Golgi apparatus by attaching oligosaccharide chains to Asn residues of proteins (Asn-X-Ser/Thr, X≠Pro) (6, 7). N-linked glycosylation facilitates proper protein folding and helps maintain protein structural stability (8, 9); N-linked glycosylation regulates the intracellular trafficking and localization of proteins (10, 11). Currently, the research on the glycosylation of tetherin mainly focuses on the human and equine, while the glycosylation mechanism of canine tetherin and its impact on antiviral function have not been fully explored. Tunicamycin, known as a N-linked glycosylation inhibitor, is a natural nucleoside antibiotic derived from actinomycetes (12, 13). Tunicamycin blocks the initial step of glycoprotein synthesis by inhibiting the transfer of N-acetylglucosamine 1-phosphate (GlcNAc-1-P) from UDP-GlcNAc to dolichol phosphate (dolichol-P). The inhibition of N-linked glycosylation leads to the accumulation of unfolded proteins in the endoplasmic reticulum and induces endoplasmic reticulum stress (14, 15).

Canine influenza virus (CIV) belongs to the family *Orthomyxoviridae* and the genus *AlphaInfluenzavirus*. CIV is a segmented, single-stranded, negative-sense RNA virus (16). CIV is a respiratory pathogen that can cause fever, runny nose, cough, and depression in canines (17). In 2004, CIV was first identified and isolated from racing canines in Florida, USA (18). CIV included two subtypes, H3N2 CIV and H3N8 CIV (19, 20). The H3N2 CIV was produced by the cross-species transmission of avian influenza virus, while the H3N8 CIV was produced by the cross-species transmission of the equine influenza virus, both of which are capable of sustained circulation in canine populations (19, 20). The epidemic of CIV poses a serious threat to the health of canines.

Tetherin has a certain restriction effect on some influenza A virus (IAV), but the restriction effect is strain-specific (21). Previous studies have shown that canine tetherin is able to restrict the release of H3N2 CIV, thereby preventing CIV from infecting the healthy cells (22, 23). However, it is unclear how the post-translational glycosylation modifications affect the antiviral function of canine tetherin. In this study, we investigated the effects of post-translational glycosylation modifications of canine tetherin on its expression, subcellular localization, and antiviral activity by using tetherin glycosylation site mutants and N-linked glycosylation inhibitors.

## 2 Results

### 2.1 Glycosylation site prediction and expression analysis of canine tetherin

**Figure 1:**
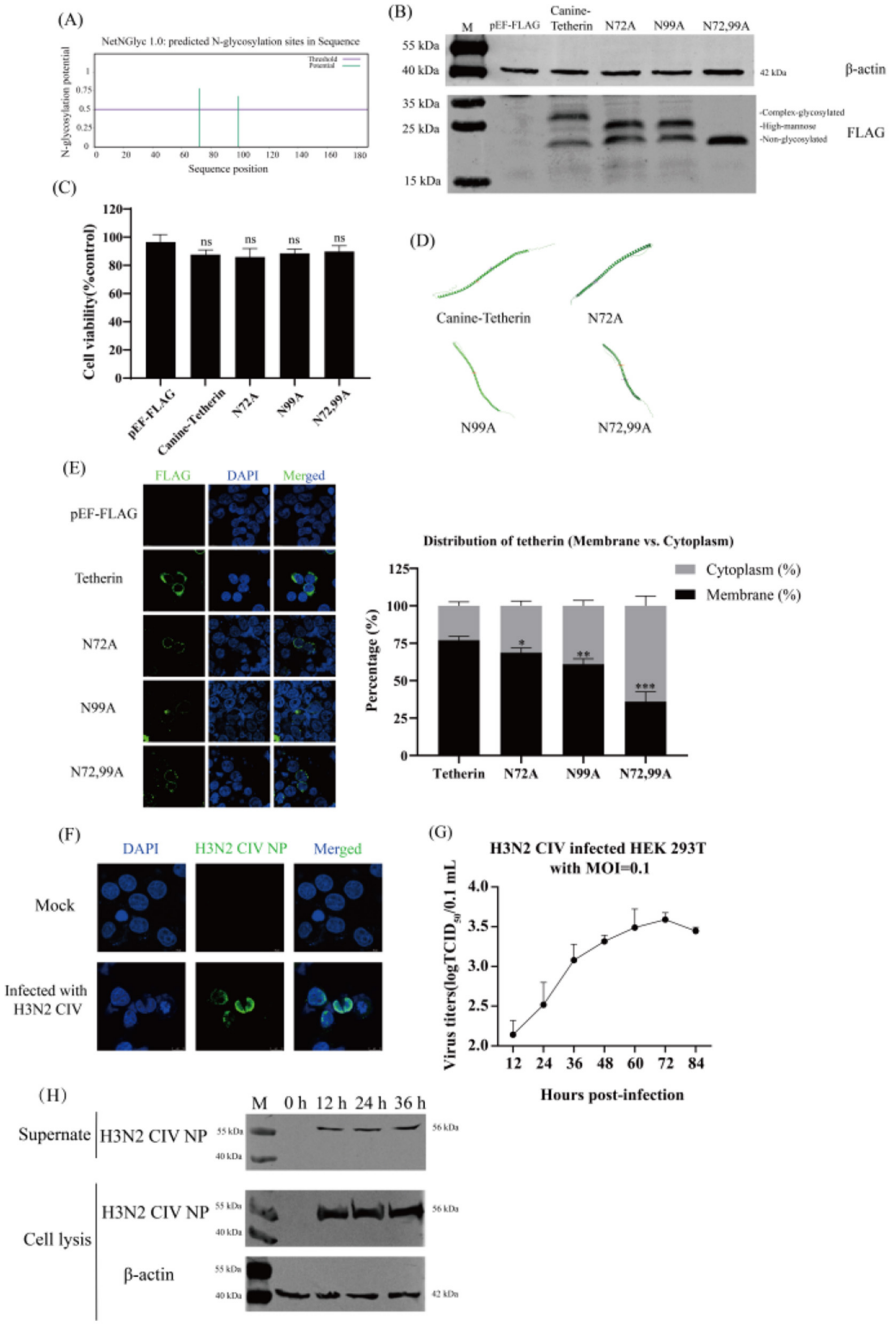
The prediction of glycosylation sites of the canine tetherin and the H3N2 CIV infection of HEK 293T cells. **(A)** Prediction of canine tetherin glycosylation sites. The amino acid sequence of canine tetherin was submitted to NetNGlyc-1.0 for glycosylation site prediction. NetNGlyc-1.0 (NetNGlyc 1.0–DTU Health Tech - Bioinformatic Services). The eukaryotic expression plasmids Canine-Tetherin, N72A, N99A, and N72,99A were transfected into HEK 293T cells. **(B)** Expression of canine tetherin protein and its glycosylation site mutants. Western blot was performed 24 h post-transfection. Lane 1: M, protein marker; lane 2: pEF-FLAG vector; lane 3: Canine-Tetherin; lane 4: N72A; lane 5: N99A; lane 6: N72,99A. **(C)** Cell viability assay. The CCK-8 assay was used to determine the viability of HEK 293T cells. **(D)** 3D structure modeling of canine tetherin and glycosylation site mutants. AlphaFold Server was used to predict the 3D structure of canine tetherin and glycosylation site mutants (https://alphafoldserver.com/). **(E)** Subcellular localization of canine tetherin and glycosylation site mutants. The distribution of each protein was observed and quantitatively analyzed. Scale bar = 10 μm. The 293T cells were infected with H3N2 CIV at a MOI of 0.1. After 1 h of incubation, the medium was replaced with DMEM containing 0.2% BSA and 0.5 μg/ml TPCK-trypsin. **(F)** H3N2 CIV infected HEK 293T cells. IFA was performed 24 h post-infection. Blue indicates DAPI, and green indicates H3N2 CIV NP. Scale bar = 10 μm. **(G)** One-step growth curve assay. The viral titers were determined by TCID_50_ method. **(H)** Western blot analysis. The proteins in the cells were extracted by using RIPA lysis buffer. Lane 1: M, protein marker; lane 2: 0 h; lane 3: 12 h; lane 4: 24 h; lane 5: 36 h. Three independent experiments were carried out separately. A *p*-value of <0.05 was considered to indicate significance (ns, not significant, *p* > 0.05; *p < 0.05; **p < 0.01; ***p < 0.001).

The amino acid sequence of canine tetherin was submitted to NetNGlyc1.0. The results showed that the canine tetherin contained two potential glycosylation sites, N72 and N99 (the threshold in the figure is 0.5, beyond which it is considered a potential glycosylation site; Figure 1A and Supplementary Figure S1). The prediction results from GlycoEP and Musite were shown in Supplementary Figure S1. The canine tetherin and mutants were expressed in Human embryonic kidney (HEK) 293T cells, respectively. Due to the glycosylation pattern, canine tetherin shows three bands (complex-glycosylated tetherin, high-mannose glycosylation, and non-glycosylated tetherin) in western blot. N72A and N99A show two bands (high-mannose glycosylation, and non-glycosylated tetherin), while N72,99A only show one band (non-glycosylated tetherin; Figure 1B). Cell viability assays showed that the viability of all experimental groups remained close to 100% after transfection with canine tetherin and mutants (Figure 1C), indicating that under the conditions of this experiment, the cell viability of HEK 293T cells was not significantly affected.

Canine tetherin and its mutants exhibited similar folding patterns (α-helix or β*-*sheet) and three-dimensional conformations (Figure 1D), indicating that mutations at the glycosylation sites did not affect the core structural framework of the tetherin. Canine tetherin was localized on the cell membrane. The N72A and N99A mutants were primarily localized on the cell membrane, whereas the N72,99A was localized not only on the membrane but also partially in the cytoplasm (Figure 1E). These results suggest that mutations at the glycosylation sites alter the subcellular localization of canine tetherin.

### 2.2 H3N2 CIV infects HEK 293T cells

After H3N2 CIV infection HEK293T cells, the intracellular distribution of H3N2 CIV was detected by IFA. The NP (green) was mainly distributed around the nucleus (Figure 1F), indicating that H3N2 CIV NP was expressed intracellularly. The results of the one-step growth curve of the virus showed that the titer of H3N2 CIV increased with time and reached the peak (3.5 logTCID_50_/0.1 ml) at 72 h of infection (Figure 1G), indicating that H3N2 CIV effectively replicated and released within the cells. Western blot results showed that H3N2 CIV NP in the supernatant and cells began to appear 12 h after infection and gradually increased with the prolongation of infection time (Figure 1H). These results indicate that H3N2 CIV effectively infects HEK 293T cells, and HEK 293T cells can be used to study the infection and replication mechanism of H3N2 CIV.

### 2.3 Effect of canine tetherin and mutants on the replication of H3N2 CIV

**Figure 2:**
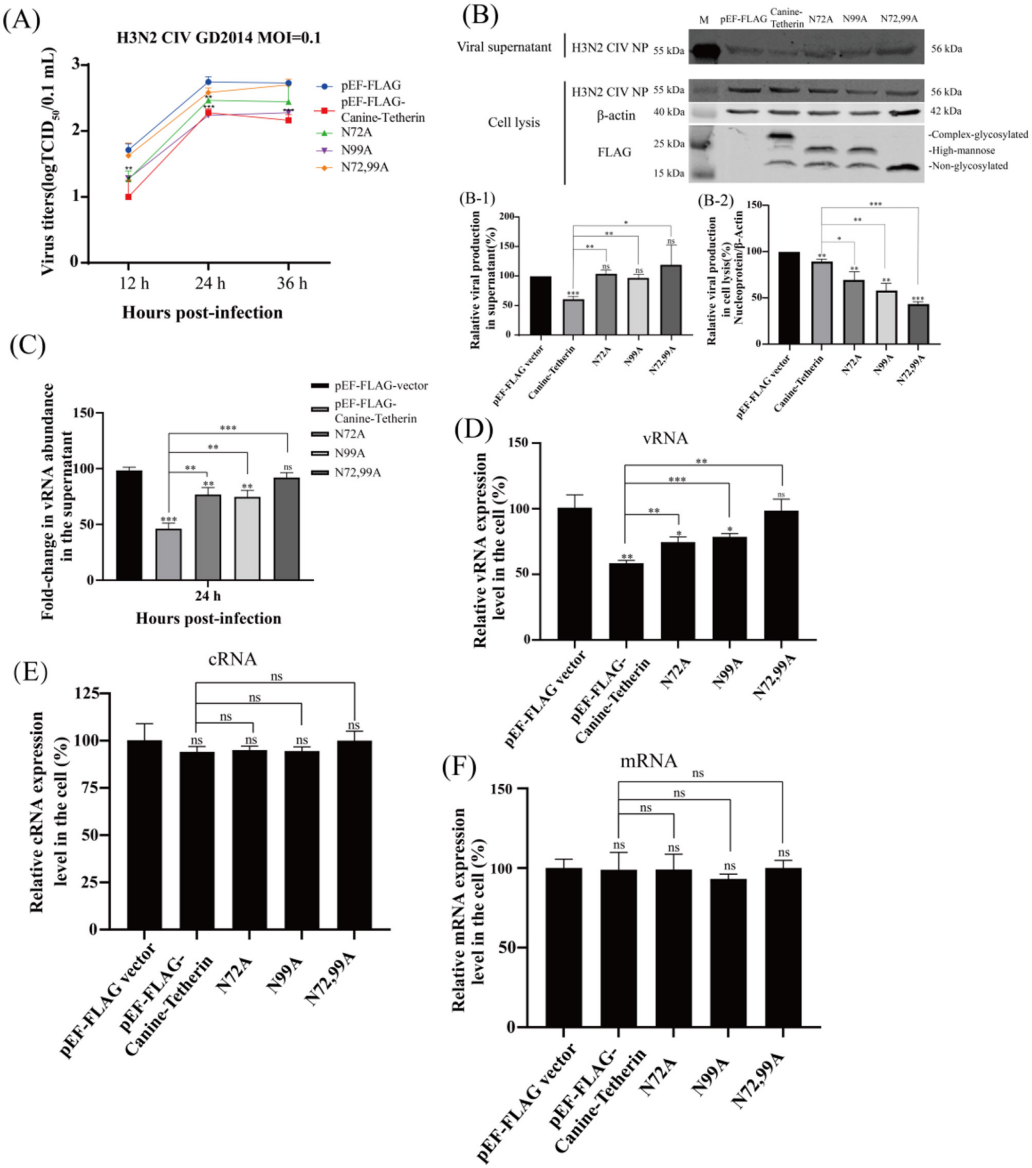
Investigating the effect of canine tetherin and its glycosylation site mutants on H3N2 CIV replication. HEK 293T cells were transfected with pEF-FLAG, Canine-Tetherin, N72A, N99A, N72,99A, and after 24 h of transfection, the cells were infected with H3N2 CIV with an MOI of 0.1, and the viral supernatant was collected. **(A)** Effect of canine tetherin and glycosylation site mutations on H3N2 CIV replication. Viral titer in the supernatant was determined using the TCID_50_ method. **(B)** Western blot analysis of the relative expression levels of the virus. RIPA lysis buffer was used to extract proteins from cells. Lane 1: M, protein marker; lane 2: pEF-FLAG vector; lane 3: Canine-Tetherin; lane 4: N72A; lane 5: N99A; lane 6: N72,99A. **(B-1)** Analysis of the gray value of H3N2 CIV NP in the supernatant. The NP expression level of pEF-FLAG was set to 100%. **(B-2)** Relative expression of H3N2 CIV NP in cell lysates. The ratio of pEF-FLAG NP to β-actin was set to 100%. Three independent experiments were carried out separately. **(C–F)** The relative expression level of CIV RNA in infected-cells was detected by RT-qPCR. **(C)** The detection of H3N2 CIV vRNA in the supernatant at 24 h post-infection by RT-qPCR. **(D)** The relative expression level of CIV vRNA in cells. **(E)** The relative expression level of CIV cRNA in cells. **(F)** The relative expression level of CIV mRNA in cells. Statistical significance was determined using the conventional Student's *t*-test and calculated with GraphPad Prism Software 6. A *p*-value of <0.05 was considered to indicate significance (*p < 0.05; **p < 0.01; ***p < 0.001). The ns represents no significance.

The viral titers of H3N2 CIV at different time points were determined by the TCID_50_ method. The results showed that, compared to the control group, canine tetherin significantly reduced the titer of H3N2 CIV. The single-site mutants (N72A and N99A) were still able to restrict viral replication, but the restriction effects were weakened. The viral titer in the N72,99A group was higher than that in the canine tetherin group, but still lower than in the control group (Figure 2A). The expression of H3N2 CIV protein was detected by western blot. The results showed that, compared to the control group, the tetherin group exhibited a significant decrease in NP expression. In contrast, the glycosylation mutant groups (N72A, N99A, and N72,99A) showed increased NP expression, with the N72,99A group displaying the most pronounced recovery (Figure 2B). The amount of virus released in the mutant group in the supernatant was higher than in the tetherin group, but still lower than in the control group (Figure 2B-1). The levels of viral protein in the tetherin group were the lowest in the cell lysate, and the levels of viral protein in the mutant group were higher than those in the tetherin group, but still lower than those in the control group (Figure 2B-2).

The viral RNA (vRNA) levels in the viral supernatant were analyzed by RT-qPCR. The results showed that canine tetherin significantly reduced the level of H3N2 CIV vRNA (^**^p < 0.01), and the mutant group partially restored the vRNA level (Figure 2C). Similarly, the intracellular vRNA levels of H3N2 CIV were significantly reduced in the tetherin group, while the mutant groups showed higher vRNA levels than the tetherin group but still lower than those in the control group (Figure 2D), indicating a weakened restriction effect of the mutants. Canine tetherin exerts a certain restrictive effect on intracellular complementary RNA (cRNA), although the extent is weaker compared to its effect on vRNA. The single glycosylation site mutants (N72A and N99A), as well as the double mutant (N72,99A), were able to partially restore cRNA levels, with the most notable restoration observed in the N72,99A group (Figure 2E). The messenger RNA (mRNA) levels changed slightly, and there was no significant difference among the groups (Figure 2F). These results indicate that canine tetherin restricts the replication and release of H3N2 CIV, but mutations at the glycosylation sites reduce or even abolish its ability to restrict H3N2 CIV replication.

### 2.4 Effect of tunicamycin treatment on the ability of canine tetherin to restrict H3N2 CIV replication

**Figure 3:**
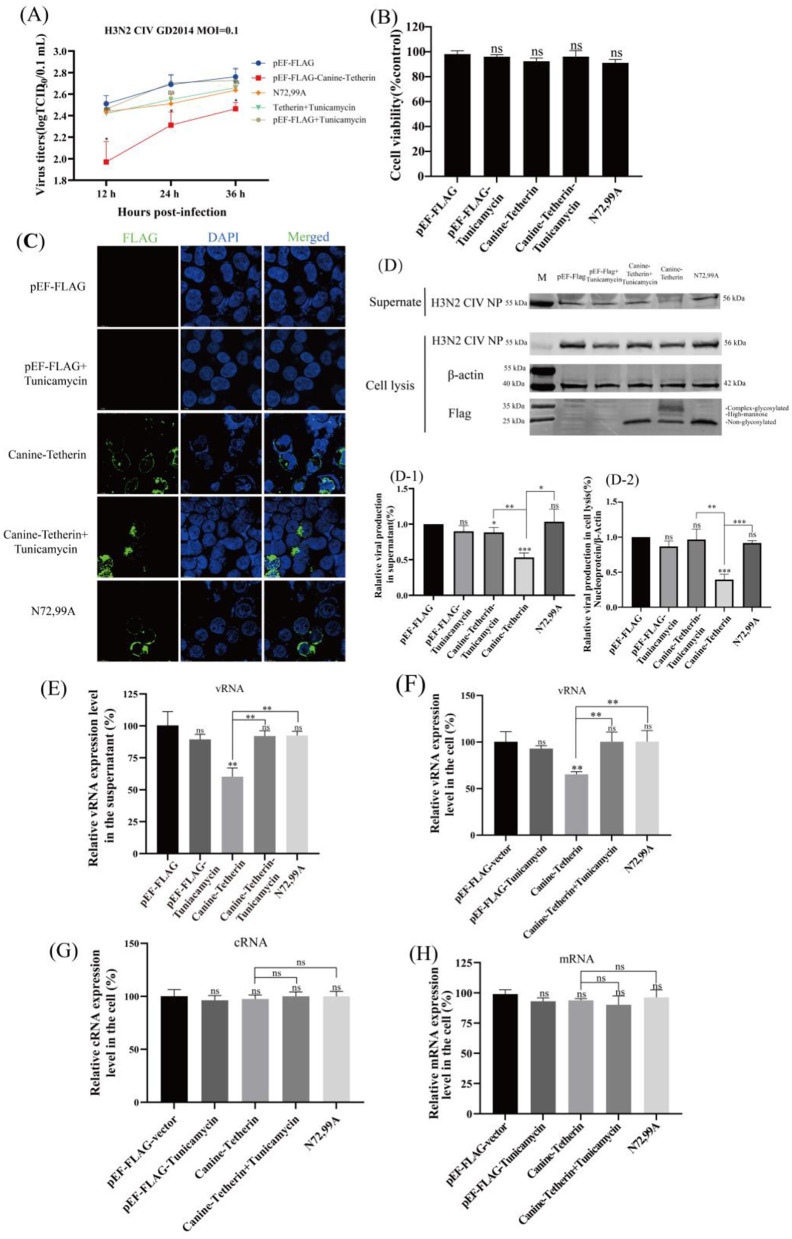
Investigating the effect of glycosylation inhibitors on the ability of canine tetherin to restrict CIV replication. HEK 293T cells were transfected with pEF-FLAG, Canine-Tetherin and N72,99A respectively. Eight hours after transfection, 1% FBS DMEM containing 1 μg/ml tunicamycin was added to the Canine-Tetherin group. After transfection for 24 h, H3N2 CIV was inoculated at a MOI of 0.1. The viral supernatant was collected. **(A)** Effect of canine tetherin and glycosylation site mutations on H3N2 CIV replication. **(B)** The cell viability of HEK 293T treated with tunicamycin. **(C)** The subcellular localization of canine tetherin treated with tunicamycin. Blue represents DAPI and green represents Alexa Fluor^®^ 488. Scale bar = 10 μm. **(D)** Western blot analysis of the relative expression levels of the virus. Lane 1: M, protein marker; lane 2, pEF-FLAG; lane 3, pEF-FLAG-Tunicamycin; lane 4, Canine-Tetherin+Tunicamycin; lane 5, Canine-Tetherin; lane 6, N72,99A. **(D-1)** Analysis of the gray value of H3N2 CIV NP in the supernatant. The NP expression level of pEF-FLAG was set to 100%. **(D-2)** Relative expression of H3N2 CIV NP in cell lysates. The ratio of pEF-FLAG NP to β-actin was set to 100%. Three independent experiments were carried out separately. **(E–H)** The relative expression level of CIV vRNA, cRNA and mRNA in tunicamycin-treated HEK 293T cells was detected by RT-qPCR. **(E)**. The detection of H3N2 CIV vRNA in the supernatant at 24 h post-infection by RT-qPCR. **(F)** The relative expression level of CIV vRNA in cells. **(G)** The relative expression level of CIV cRNA in cells. **(H)** The relative expression level of CIV mRNA in cells. Statistical significance was determined using the conventional Student's *t*-test and calculated with GraphPad Prism Software 6. A *p*-value of <0.05 was considered to indicate significance (*p < 0.05; **p < 0.01; ***p < 0.001). The ns represents no significance.

HEK 293T cells were transfected with Canine-Tetherin or treated with tunicamycin. The virus titer in the tetherin group decreased significantly, the virus titer in the N72,99A groups increased, and the H3N2 CIV titer in the tunicamycin treatment group also increased (Figure 3A), indicating that by interfering with the glycosylation modification of canine tetherin, the antiviral effect of tetherin was weakened. The results of cell viability detection showed that there was no significant difference in cell viability among the treatment groups (Figure 3B). Immunofluorescence staining analysis showed that canine tetherin was localized on the cell membrane, while N72,99A and tunicamycin treatment altered the localization of canine tetherin, making it more dispersed within the cytoplasm (Figure 3C). Western blot results showed that the NP expression in the supernatant decreased in canine tetherin group, and the NP expression level in the N72,99A and Tunicamycin treatment groups was higher, which was similar to that in the control group (Figures 3D, D-1). Tetherin group NP expression in cell lysates showed stronger intracellular retention, whereas tunicamycin treatment or N72,99A mutations resulted in decreased NP levels in cell lysates (intracellular) and increased NP expression levels in supernatants (Figures 3D, D-2). Wild-type canine tetherin effectively restricts the release of H3N2 CIV into the cell, while treatment with tunicamycin or N72,99A impairs this effect (increased viral release efficiency).

The vRNA levels in the tetherin group in the supernatant and intracellular were significantly decreased (^**^p < 0.01), while the vRNA levels in the tunicamycin treatment and N72,99A groups were restored (Figures 3E, F). The cRNA level slightly decreased in the tetherin group, while cRNA levels recovered in the tunicamycin treatment group and the N72,99A group, and there was no significant difference compared with the control group (Figure 3G). There was no significant difference in viral mRNA levels between groups (Figure 3F). The above results indicate that the wild-type canine tetherin effectively restricts the replication and release of H3N2 CIV by relying on the glycosylation function. Mutation of glycosylation sites or treatment with N-linked glycosylation inhibitors alters the membrane localization and function of tetherin, leading to a reduction in its antiviral activity.

### 2.5 Colocalization analysis of canine tetherin and glycosylated mutants with H3N2 CIV proteins

**Figure 4:**
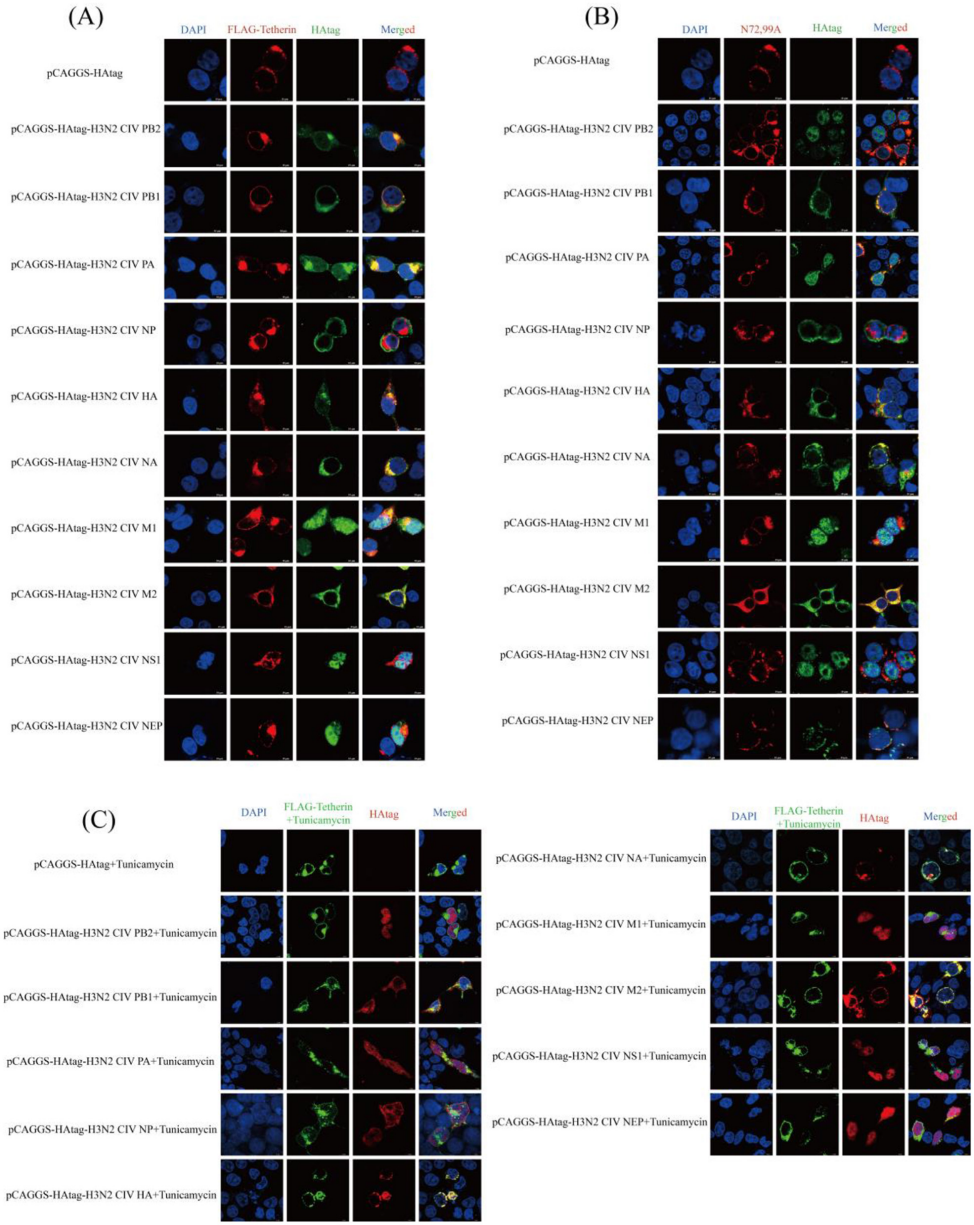
Cellular co-localization analysis of canine tetherin and its glycosylation site mutants with H3N2 CIV proteins. **(A)** Cellular localization between canine tetherin and H3N2 CIV proteins. **(B)** Cellular localization between N72,99A and H3N2 CIV proteins. **(C)** Cellular co-localization of canine tetherin and H3N2 CIV proteins after tunicamycin treatment. Blue represents DAPI, red represents Alexa Fluor^®^ 594, and green represents Alexa Fluor^®^ 488. Scale bar = 10 μm.

Laser scanning confocal microscopy revealed co-localization signals (yellow) between wild-type canine tetherin and H3N2 CIV proteins (PB2, PB1, PA, HA, NA, M1, and M2; Figure 4A), suggesting potential spatial proximity or direct interactions among these proteins. The colocalization signal between the glycosylation site mutant (N72,99A) and the H3N2 CIV (PB2, PB1, PA, and HA) proteins is attenuated, with little coincidence with NA and M1 (Figure 4B). The colocalization of canine tetherin with H3N2 CIV protein after glycosylation inhibitor treatment was similar to that of N72,99A (Figure 4C). These results indicate that the loss of glycosylation in canine tetherin impairs its ability to spatially co-localize with H3N2 CIV proteins.

## 3 Discussion

Tetherin is an interferon-induced innate immune restriction factor (2, 24). Tetherin physically binds virions to the surface of the cell membrane through its unique topology (24). N-linked glycosylation is an important step in the post-translational modification of proteins. The proper glycosylation modifications help proteins perform their biological functions. This study investigated the effect of canine tetherin and its glycosylation modifications on the replication of H3N2 CIV, revealing the critical role of N-linked glycosylation in the antiviral activity of canine tetherin.

Two potential N-linked glycosylation sites of canine tetherin at positions N72 and N99 were identified by bioinformatics software (Figures 1A, Supplementary Figure S1). The mutations at N72 and N99 sites in canine tetherin altered their glycosylation status, but did not affect their translation and expression (Figure 1B). Subsequently, the three-dimensional structure prediction of canine tetherin and mutants showed that the mutation of the glycosylation site did not change the overall 3D structure conformation of tetherin (Figure 1D). Previous studies have shown that the loss of glycosylation sites in corin protein leads to reduced distribution on the surface of HEK 293T cells (25). To investigate whether mutations at the glycosylation sites of canine tetherin affect its membrane localization, the tetherin mutants were overexpressed in cells, respectively. The glycosylation site mutant (N72,99A) was different in cell localization, and in addition to being localized on the cell membrane, a diffuse distribution was detected inside the cell (Figure 1E), suggesting that the mutation of the glycosylation site of canine tetherin affect its anchoring on the membrane. This is similar to equine tetherin, where mutations at the glycosylation sites result in the accumulation of equine tetherin in the cytoplasm and a significant reduction of the protein localized on the plasma membrane (1).

The expression of viral proteins in cells and the efficient replication and release of CIV in HEK 293T cells were verified (Figures 1F–H), which indicates that HEK 293T cells can be used to study the infection and replication mechanism of H3N2 CIV. Bai et al. (1) reported that mutations at the N-linked glycosylation sites of equine tetherin led to a significant increase in the release of equine infectious anemia virus (EIAV) particles from cells. However, whether this function is conserved in canine tetherin remains to be investigated. When individual glycosylation sites were mutated (N72A and N99A), the H3N2 CIV titer increased compared to the wild-type tetherin group. When all glycosylation sites of canine tetherin were mutated, the viral titer increased markedly, though it remained lower than that of the control group (Figure 2A). Mutation of the glycosylation sites in canine tetherin partially restored the expression of H3N2 CIV proteins, thereby weakening its restrictive effect on H3N2 CIV replication (Figure 2B), which is consistent with the findings reported by Bai et al. The results of nucleic acid changes suggest that the glycosylation modification of tetherin affects the levels of H3N2 CIV vRNA and cRNA, but has little effect on viral mRNA (Figures 2D–F). Mutations in the glycosylation site of canine tetherin impairs the ability of tetherin to restrict H3N2 CIV. These results indicate that the impact of tetherin N-linked glycosylation on its antiviral activity is evolutionarily conserved.

Given that tunicamycin globally inhibits N-linked glycosylation, its effects may extend beyond tetherin alone, potentially influencing the expression, trafficking, or function of other glycoproteins. Therefore, the observed phenotypes should be interpreted in conjunction with the experimental results from canine tetherin glycosylation mutants. After treatment with tunicamycin, the canine tetherin glycosylation modification was inhibited, and western blot showed only a non-glycosylated form (Figure 3D). After tunicamycin treatment, tetherin is more dispersed in the cytoplasm (Figure 3C). Similar to N72,99A, the viral titer in the tunicamycin-treated group was significantly higher than that in the non-treated and control groups (Figure 3A), and was further verified in terms of viral protein expression and viral vRNA levels (Figures 3D–H).

Tetherin exhibits co-localization with multiple H3N2 CIV proteins (Figure 4A), suggesting that it may exert antiviral effects through various mechanisms. For instance, Tetherin may interfere with the assembly of the CIV polymerase complex, impede the processing and trafficking of viral surface glycoproteins (HA and NA), or capture viral membrane proteins (M1 and M2) to restrict viral budding. However, when glycosylation is disrupted (Figures 4B, C), the intracellular localization of tetherin is altered, preventing it from targeting regions where viral proteins are active. This leads to a significant reduction in its co-localization with viral proteins and a consequent loss of its restriction capacity. These results clarify the central role of glycosylation of canine tetherin in maintaining its membrane localization, mediating interaction with viral proteins, and exerting antiviral functions. The antiviral activity of tetherin depends not only on its structural properties, but also on its post-translational modifications. This phenomenon also implies that the virus may evade host immunity by interfering with host glycosylation processes. Conversely, maintaining the glycosylation of tetherin may serve as an important strategy for the host to enhance innate immune defense and restrict viral infection.

In conclusion, canine tetherin has anti-influenza virus activity, and glycosylation modification is the structural basis for maintaining the normal localization of canine tetherin and its functional binding to proteins, and regulating the glycosylation status of canine tetherin affects its antiviral activity. This provides a theoretical basis for the future development of anti-influenza strategies for small molecules targeting glycosylation modifications.

## 4 Materials and methods

### 4.1 Viruses, cells and drugs

The H3N2 CIV strain A/canine/Guangdong/02/2014 (GD/2014) was isolated from free-range canine sick with respiratory diseases in Guangdong Province, China, by the Guangdong Technological Engineering Research Center for Pet, College of Veterinary Medicine, South China Agricultural University. The sample collection and virus isolation were approved by the Ethics Committee of the Laboratory Animal Center of South China Agricultural University. The H3N2 CIV GD/2014 was propagated in 9- to 11-day-old specific pathogen-free embryonated chicken eggs and stored at −80°C.

Human embryonic kidney (HEK) 293T cells and Madin–Darby Canine Kidney (MDCK) cells were grown in DMEM (G4515, Servicebio, Wuhan, China) with 10% fetal bovine serum (FBS, A5256701, Gibco, GrandIsland, USA) and 1% penicillin and streptomycin (C0222, Beyotime, Shanghai, China). All work with human cells was performed under appropriate biosafety protocols to mitigate zoonotic risk.

Tunicamycin (SC0393, Beyotime, Shanghai, China) was purchased from Beyotime. The effective concentration of tunicamycin was selected based on existing literature and applied in subsequent experiments (26).

### 4.2 Prediction of glycosylation sites and 3D structure of proteins

The amino acid sequence of canine tetherin was submitted to NetNGlyc1.0 to predict potential glycosylation sites in canine tetherin. GlycoEP and Musite were used to validate the predicted N-linked glycosylation sites of canine tetherin.

The amino acid sequences of canine tetherin and glycosylation site mutants were submitted to AlphaFold. The predicted models were opened using PyMOL software, labeled and displayed with the corresponding amino acid residues.

Mutate the asparagine (N) residues at positions 72 and 99 of canine tetherin to alanine (A). The eukaryotic expression plasmids of canine tetherin (pEF-FLAG-Canine-Tetherin) and glycosylation site mutants (N72A, N99A, and N72,99A) were constructed.

### 4.3 CCK-8 assay

The CCK-8 (C0037, Beyotime, Shanghai, China) assay was performed to determine the effect of each eukaryotic expression plasmid on the activity of HEK 293T cells. The CCK-8 assay was carried out according to the standard protocol.

### 4.4 IFA and confocal microscopy

Co-transfect HEK 293T cells with the plasmids Canine-Tetherin, N72,99A, and pCAGGS-HAtag-H3N2 CIV, respectively. After 24 h of transfection, the medium was discarded and the cells were washed with PBS, and the cells were fixed with 4% paraformaldehyde (PFA; BL539A, Biosharp, Hefei, China) for 10 min at 4°C. To ensure accurate localization of the membrane protein, 293T cells were not subjected to membrane permeabilization during the staining procedure. Three percent of BSA (A1933, Sigma-Aldrich, Burlington, MA, USA) in PBS was used at room temperature to block non-specific binding. Mouse monoclonal anti-FLAG antibody (F3165, Sigma-Aldrich, Burlington, MA, USA) and rabbit HA Tag Recombinant antibody (81290, Proteintech, Wuhan, China) were each diluted 1:500 and incubated with the cells overnight at 4°C. The cells were washed with PBS. Goat anti-mouse IgG H&L (Alexa Fluor^®^ 594; ab150116, Abcam, Cambridge, UK) and Goat Anti-Rabbit IgG H&L (Alexa Fluor^®^ 488; ab150077, Abcam, Cambridge, UK) were diluted 1:500 and incubated for 1 h at room temperature, respectively. The cells were washed with PBS and nuclear staining was performed using DAPI (C1006, Beyotime, Shanghai, China), and then observed under a confocal microscope. ImageJ software was used to quantify the membrane vs. cytoplasmic distribution of the protein.

### 4.5 Assessment of mutations in the glycosylation sites of tetherin on its antiviral activity

#### 4.5.1 Viral titer determination

HEK 293T cells were transfected with N72A, N99A, and N72,99A and were infected with the H3N2 CIV GD2014 strain at a multiplicity of infection (MOI) of 0.1 at 24 h post-transfection. After incubation for 1 h, the virus was discarded and the cells were washed with PBS. DMEM containing TPCK-trypsin at a final concentration of 0.5 μg/ml and 0.2% BSA was added into the plates. Two hundred microliters of the supernatant from the cell plates was collected at 12 h intervals to determine their viral titers. The titer of virus in the supernatant was determined using the 50% Tissue culture infective dose (TCID_50_) method. MDCK cells were prepared and coated into 96-well plates. The collected supernatant was performed serially 10-fold dilutions in DMEM containing 1% FBS and 0.5 μg/ml TPCK-trypsin. One hundred microliters of diluted virus solution was added to each well. Five replicates were set for each dilution, and negative controls were set in the last two columns. The medium in the 96-well plate was discarded for 48 h post-incubation, and the IFA was performed. The cells were incubated with rabbit-derived H3N2 CIV NP polyclonal antibody, and the goat anti-rabbit IgG H&L (Alexa Fluor^®^ 488) was used as fluorescent secondary antibody. TCID_50_ was calculated using the Reed–Muench method.

#### 4.5.2 Reverse transcription polymerase chain reaction (RT–qPCR) analysis

The relative expression levels of CIV RNA in intracellular and supernatants were determined using RT-qPCR. The viral RNA (vRNA) refers to the negative-sense viral genomic RNA. The complementary RNA (cRNA) is the positive-sense complementary RNA synthesized from vRNA, acting as an intermediate during replication to generae new vRNA. The messenger RNA (mRNA) is the positive-sense messenger RNA transcribed from vRNA, which was used for translation of viral proteins by the host machinery. The RNA in the supernatant and HEK 293T cells were extracted using Trizol method. HiScript III 1st Strand cDNA Synthesis Kit (+gDNA wiper; R312, Vazyme, Nanjing, China) was used to product the cDNA. The primer IAV-vRNA was used to produce the cDNA for CIV vRNA detection. The IAV-cRNA was used to produce the cDNA for CIV cRNA detection. The oligo(dT) primer (A212, GenStar, Beijing, China) was used to generate cDNA for CIV mRNA. RT-qPCR was used to detect the relative expression levels of CIV vRNA, cRNA, and mRNA. The primers for RT-qPCR were listed in the Supplementary Table S1.

#### 4.5.3 Western blot analysis

HEK 293T cells were transfected with pEF-FLAG, pEF-FLAG-Canine-Tetherin, N72A, N99A, and N72,99A. The cells were infected with H3N2 CIV GD2014 at MOI of 0.1 at 24 h post-transfection. After incubation for 1 h at 37°C in 5% CO_2_, the viral was discarded and the cells were washed twice with PBS. Subsequently, the infected cells were cultured with DMEM containing 0.2% BSA and 0.5 μg/ml TPCK-trypsin. After 24 h of infection, the viral protein in the supernatant and the total protein in cells were extracted, respectively. For the extraction of viral proteins from the supernatant, centrifuge the supernatant from the 6-well plates to remove cellular debris, and transfer equal volumes of the clarified supernatant into 1.5 ml microcentrifuge tubes. One-fourth volume of Omni-Easy™ Instant Protein Loading Buffer (Denaturing, Reducing, 5×; LT101, YEASEN, Shanghai, China) was added to each tube. After thorough mixing, the samples were heated in a 95°C water bath for 5 min and then subjected to subsequent western blot analysis. Western blot was used to analyze viral protein relative expression levels. The ImageJ software was used for the analysis of protein gay values.

### 4.6 The effect of glycosylation inhibitors on the antiviral activity of tetherin

#### 4.6.1 Subcellular localization of canine tetherin treated with tunicamycin

The pEF-FLAG-Canine-Tetherin was transfected into HEK 293T cells, the medium was replaced with the fresh DMEM containing a final concentration of 1 μg/ml of tunicamycin at 8 h post-transfection, and the total protein was extracted after 24 h post-transfection or the cells were performed with IFA.

#### 4.6.2 Viral titer determination, RT-qPCR and western blot analysis

The transfected HEK 293T cells were treated with tunicamycin and infected with H3N2 CIV GD2014 strain at an MOI of 0.1. The viral titer of CIV in the supernatants was determined by TCID_50_ method.

RT-qPCR was used to detect the relative expression level of CIV RNA in the supernatants and the cells. The protein in the supernatant and the cells was extracted. Western blot was performed.

## Data Availability

The original contributions presented in the study are included in the article/Supplementary material, further inquiries can be directed to the corresponding authors.
