# Correction: Glycosylation of canine tetherin is essential for its antiviral activity against H3N2 canine influenza virus

**DOI:** 10.3389/fvets.2026.1888429

**Published:** 2026-06-15

**Authors:** Liang Xu, Yuwen Hou, Shizhe Liu, Yixin Dai, Jiajun Ou, Shaotang Ye, Zhen Wang, Gang Lu, Shoujun Li

**Affiliations:** 1 College of Veterinary Medicine, South China Agricultural University, Guangzhou, China; 2 Guangdong Technological Engineering Research Center for Pet, Guangzhou, China

**Keywords:** tetherin, N-linked glycosylation, mutation, canine influenza virus (CIV), antiviral activity

In the original article, there was an error. The information for one research grant (Guangdong Provincial Natural Science Foundation) was erroneously omitted.

The correct funding statement reads:

## Funding

The author(s) declared that financial support was received for this work and/or its publication. This work was supported by grants from the National Natural Science Foundation of China (32172826) and Guangdong Provincial Natural Science Foundation (2023A1515012950).

The original version of this article has been updated.

