# Supplementary material for: Glycosylation of canine tetherin is essential for its antiviral activity against H3N2 canine influenza virus
Source: Front Vet Sci. 2025 Jul 23;12:1641963. doi: 10.3389/fvets.2025.1641963 (PMC12325054; doi:10.3389/fvets.2025.1641963)

Supplemental Information

Table S1 The primers used for RT-qPCR.

| Primers | Sequence (5’-3’) |
| --- | --- |
| IAV-vRNA | AGTCTTCTAACCGAGGTCGAAACGTA |
| IAV-cRNA | AACATCCACAGCACTCTGCTGTTCCT |
| qPCR-CIV-F | TCAAGTGATCCTCTCGTTATTGC- |
| qPCR-CIV-R | CACTCTGCTGTTCCTGCCGATA |
| GAPDH-human-F | AGATCCCTCCAAAATCA AGTGG |
| GAPDH-human-R | GGCAGAGATGATGACCCTTTT |

Figure S1. N-Linked Glycosylation Site Prediction of Canine Tetherin via GlycoEP and Musite. The amino acid sequence of canine tetherin was submitted to GlycoEP (A) and Musite (B) for the prediction of N-linked glycosylation sites. The threshold is 0.5, beyond which it is considered a potential glycosylation site GlycoEP: <https://webs.iiitd.edu.in/raghava/glycoep/>. Musite: <https://www.musite.net/>.


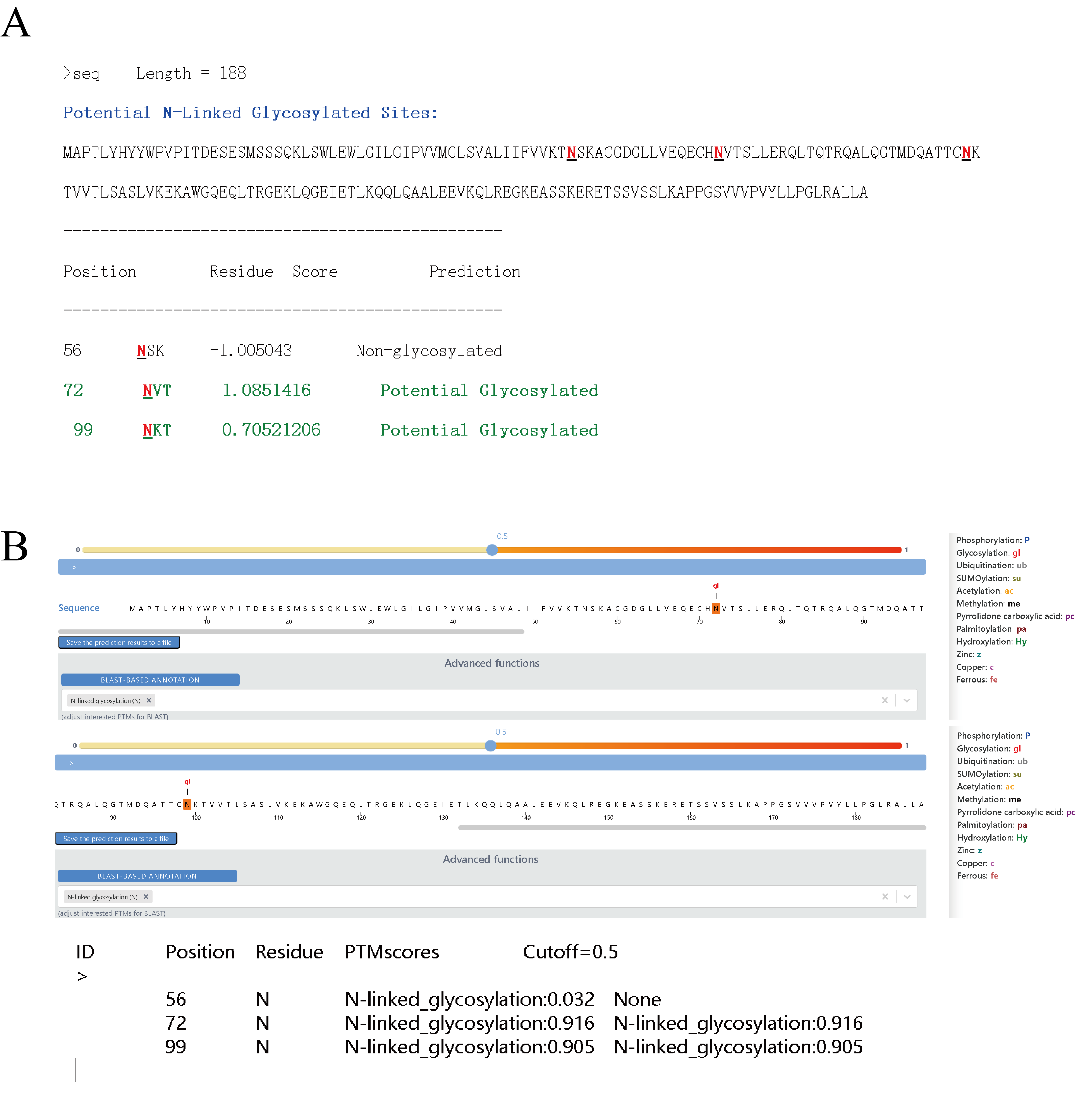

Supplement: Supplementary file 1 [file Table_1.docx]
